# Immunogenicity of a Bivalent Non-Purified Recombinant Vaccine against Botulism in Cattle

**DOI:** 10.3390/toxins10100381

**Published:** 2018-09-20

**Authors:** Clóvis Moreira, Marcos R. A. Ferreira, Carlos E. P. da Cunha, Rafael A. Donassolo, Paula F. Finger, Gustavo M. S. G. Moreira, Denis Y. Otaka, Loise A. de Sousa, José D. Barbosa, Ângela N. Moreira, Felipe M. Salvarani, Fabricio R. Conceição

**Affiliations:** 1 Centro de Desenvolvimento Tecnológico, Universidade Federal de Pelotas, Pelotas, Rio Grande do Sul 96160-000, Brazil; clovismoreirajr@live.com (C.M.J.); marcosferreiravet@gmail.com (M.R.A.F.); cpouey@gmail.com (C.E.P.d.C.); rafaeldonassolo@hotmail.com (R.A.D.); paulaafinger@hotmail.com (P.F.F.); moreira.gmsg@gmail.com (G.M.S.G.M.); angelanmoreira@yahoo.com.br (Â.N.M.); 2 Instituto de Medicina Veterinária, Universidade Federal do Pará, Castanhal, Pará 68740-910, Brazil; otaka@veterinario.med.br (D.Y.O.); loisesousa.a@gmail.com (L.A.d.S.); diomedes@ufpa.br (J.D.B.); felipems@ufpa.br (F.M.S.)

**Keywords:** *Clostridium botulinum*, vaccine, botulinum neurotoxin (BoNT), cell lysate vaccine

## Abstract

Botulism is a potentially fatal intoxication caused by botulinum neurotoxins (BoNTs) produced mainly by *Clostridium botulinum*. Vaccination against BoNT serotypes C and D is the main procedure to control cattle botulism. Current vaccines contain formaldehyde-inactivated native BoNTs, which have a time-consuming production process and pose safety risks. The development of non-toxic recombinant vaccines has helped to overcome these limitations. This study aims to evaluate the humoral immune response generated by cattle immunized with non-purified recombinant fragments of BoNTs C and D. Cattle were vaccinated in a two-dose scheme with 100, 200 and 400 µg of each antigen, with serum sampling on days 0, 56, 120, and 180 after vaccination. Animals who received either 200 or 400 μg of both antigens induced titers higher than the minimum required by the Brazilian ministry of Agriculture, Livestock and Food Supply and achieved 100% (8/8) seroconversion rate. Animals vaccinated with commercial toxoid vaccine had only a 75% (6/8) seroconversion rate for both toxins. Animals that received doses containing 400 µg of recombinant protein were the only ones to maintain titers above the required level up until day 120 post-vaccination, and to achieve 100% (8/8) seroconversion for both toxins. In conclusion, 400 µg the recombinant *Escherichia coli* cell lysates supernatant was demonstrated to be an affordable means of producing an effective and safe botulism vaccine for cattle.

## 1. Introduction

Botulism is a potentially fatal neuroparalytic disease caused by intoxication with botulinum neurotoxins (BoNTs) produced by anaerobic Gram-positive bacteria species such as *Clostridium botulinum*, *C. butyricum*, *C. barati* and *C. argentinenses*, that can be found in soil and water, as well as in the gastrointestinal tract of humans and animals [1]. BoNTs are classified into 7 serotypes (A–G) based on their antigenic characteristics [2]. Serotypes C and D, or a chimeric fusion termed C/D or D/C toxins, are involved in cattle botulism [3,4,5]. Highly lethal botulism outbreaks in livestock caused by ingestion of food or water contaminated with BoNT serotypes C and D have been reported, leading to significant economic losses [6]. In endemic countries like Australia, Brazil, South Africa, and Israel, the induction of neutralizing antibodies through vaccination is the most effective way to prevent death by botulism given the impossibility of eradicating the agent [5,7,8,9,10].

Commercially available vaccines contain formaldehyde-inactivated native BoNTs (toxoids). Although they show efficacy in generating protection in vaccinated animals, the production process shows some limitations. The detoxification process, for example, takes from 15 to 21 days and may affect BoNTs’ immunogenicity [11]. The fermentation procedure also presents high biological risk both to workers and to the environment as BoNTs are the most potent toxins known [2]. As an alternative to toxoids, the use of recombinant vaccines comprising the C-terminal portion of BoNTs serotypes C and D heavy-chain (H_C_BoNT/C and D) has been considered safe and effective in many animal species [12,13,14,15].

Recombinant vaccines for BoNT types C and D would offer several advantages over the present vaccine such as low or no toxicity, good immunogenicity, the use of non-pathogenic strains, stable and predictable yield, making it easier to scale up production and eliminating the need for detoxification steps [16]. However, purifying recombinant proteins could involve very expensive chromatography steps, which can make the process unattractive for veterinary applications [17,18].

Easily manufactured vaccines containing recombinant, non-purified H_C_BoNT/C and D were previously developed and evaluated in guinea pigs, inducing higher neutralizing antibody titers than native toxoids and similar to vaccines containing the same amount of purified proteins [19]. The present study aims to evaluate the dose-response ratio and the longevity of the humoral immune response of cattle to non-purified recombinant vaccines against botulism types C and D.

## 2. Results

### 2.1. Recombinant Vaccine Formulations are Safe for Animal Use

The sterility test resulted in no growth after a 21-day observation period, indicating either fungi or bacteria did not contaminate the formulation. All cattle were observed by a veterinary surgeon during the experiment and, as well as in innocuity test, no local or systemic adverse reactions were noticed in any animal. This supports the previous results of our group, in which non-purified H_C_BoNT/C and D were shown to be non-toxic in guinea pigs [19]. These results indicate that the non-purified recombinant vaccines mixed with aluminum hydroxide as adjuvant are safe to use in cattle.

### 2.2. Recombinant Vaccines Induce Protective Humoral Response in Cattle

The experimental design of this study included a two-dose immunization in distinct groups receiving different amounts of recombinant vaccine in order to evaluate the level and longevity of immune responses. Neutralizing antibody titers in each animal at day 56 was determined by mouse neutralization bioassay (Table 1). The recombinant formulations, consisting of cell lysate supernatant of *E. coli* expressing the toxin subunits, were capable of inducing anti-BoNT/C neutralizing mean titers of 1.25, 8.65, and 11.00 IU/mL for animals vaccinated with 100, 200, and 400 µg of each recombinant protein; and anti-BoNT/D neutralizing mean titers of 1.50, 10.25, and 13.75 IU/mL, respectively. Animals vaccinated with the commercial vaccine produced titers of 4.00 and 2.62 IU/mL against BoNT serotypes C and D, respectively. Animals in the negative control group had no detectable levels of neutralizing antibodies against either toxin.

**Figure 1:**
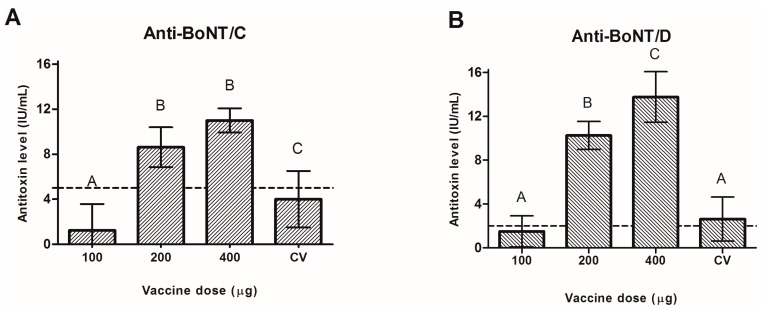
Evaluation of neutralizing antibodies against BoNT serotypes C and D in cattle at day 56 post vaccination with different concentrations of recombinant proteins or the commercial vaccine. Different capital letters (A–C) indicate statistical difference between groups (*p* < 0.001). The dashed lines represent the required minimum level of neutralizing antibodies against each BoNT as determined by MAPA (5 IU/mL and 2 IU/mL for BoNTs C and D, respectively). CV, commercial vaccine. (**A**): Neutralizing antibody mean titers against BoNT serotype C; (**B**): Neutralizing antibody mean titers against BoNT serotype D.

Animals that received doses containing 200 or 400 µg of recombinant protein achieved 100 % (8/8) seroconversion rate, while animals vaccinated with commercial toxoid vaccine had only a 75% (6/8) seroconversion rate for both toxins. When using 100 µg of recombinant protein per dose, the seroconversion rate falls to 25% and 62.5% for BoNTs C and D, respectively. Figure 1 shows the mean titers against BoNT serotype C (Figure 1A) or D (Figure 1B) to commercial vaccine (CV) and recombinant vaccine (100, 200, and 400 µg) compared by Tukey’s test (*p* < 0.001).

There was no statistical difference between mean titers of anti-BoNT/C neutralizing antibodies in animals who received either 200 or 400 μg of recombinant vaccine, but both are higher than the minimum required by the Brazilian Ministry of Agriculture, Livestock and Food Supply (MAPA) (IN23) [20]. Furthermore, these titers are statistically higher than those observed for animals who received either 100 μg of recombinant protein vaccine or the commercial toxoid vaccine (Figure 1A). Vaccination with 400 µg of H_C_BoNT/D induced significantly higher antibody titers than vaccination with 200 µg of the same protein; meanwhile, vaccination with the latter induced more antibodies than vaccination with only 100 µg, similar to the result observed in animals who received the commercial toxoid vaccine (Figure 1B). Regarding the minimum antibody level required for an anti-BoNT/D vaccine, only the vaccine containing 100 µg of recombinant protein per dose failed; all others showed a higher antibody titer, even though the commercial toxoid was barely above the minimum (2 IU/mL).

### 2.3. Recombinant Protein Concentration and Titers against Botulinum Neurotoxins (BoNTs) C and D Showed a Positive Linear Correlation

**Figure 2:**
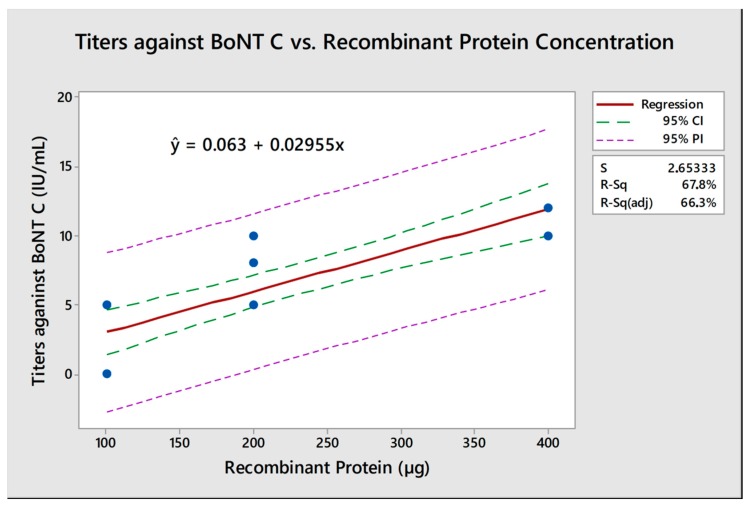
Regression equation for the administered dose of recombinant protein correlated to the neutralizing antibody titers against botulinum neurotoxin C on day 56 post vaccination. The neutralizing antibody titer in IU/mL for each animal (1–8) from each group (100, 200, and 400 µg), represented by the blue dots, were used to calculate the regression equation for this dose-response analysis. Considering the equation, it was possible to determine the minimum amount of protein necessary to achieve antibody levels above the required in all animals. In this case, 167 µg of heavy-chain BoNT (H_C_BoNT)/C was the amount calculated. CI: confidence interval; PI: prediction interval; S: standard error; R-Sq: R-squared.

**Figure 3:**
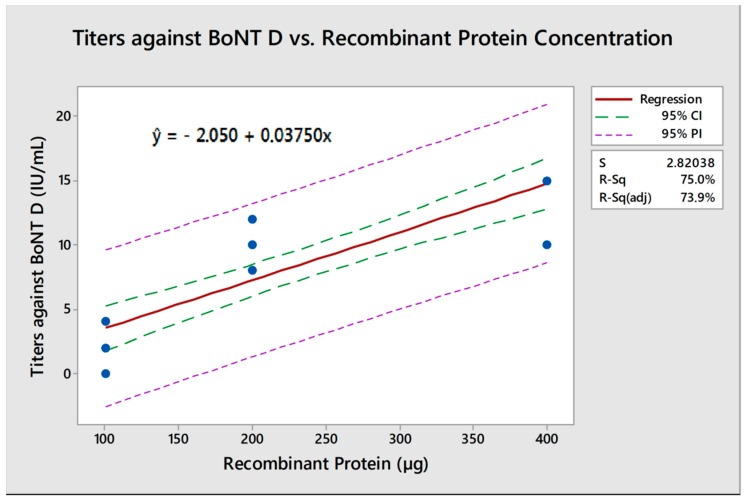
Regression equation for the administered dose of recombinant protein correlated to the neutralizing antibody titers against botulinum neurotoxin D on day 56 post vaccination. The neutralizing antibody titer in IU/mL for each animal (1–8) from each group (100, 200, and 400 µg), represented by the blue dots, were used to calculate the regression equation for this dose-response analysis. Considering the equation, it was possible to determine the minimum amount of protein necessary to achieve antibody levels above the required in all animals. In this case, 108 µg of H_C_BoNT/D was the amount calculated. CI: confidence interval; PI: prediction interval; S: standard error; R-Sq: R-squared.

According to the statistical analysis, these results showed a significative linear relation (*p* < 0.001) between recombinant protein concentration on vaccine formulations and the titers of neutralizing antibodies at day 56 post-vaccination both for BoNT/C (r_C_^2^ = 0.823) and BoNT/D (r_D_^2^ = 0.866). With the analysis obtained through an equation of regression to BoNTs C and D (Figure 2 and Figure 3), it is possible to calculate that approximately 167 µg H_C_BoNT/C and 108 µg H_C_BoNT/D is necessary to induce the minimum titers required by MAPA against BoNTs C and D on 67.8% and 75% vaccinated animals, respectively.

### 2.4. Longevity of the Immune Response in Cattle Increases with the Amount of Heavy-Chain BoNTs (H_C_BoNTs)

**Figure 4:**
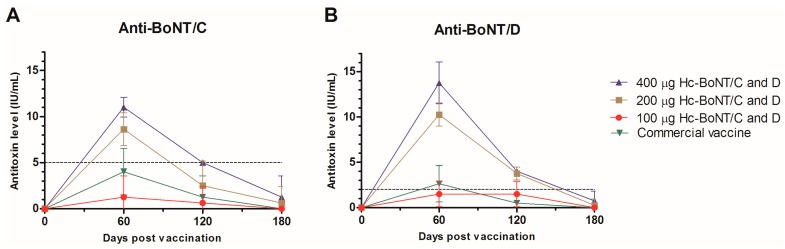
Evaluation of mean titers of neutralizing anti-BoNT serotypes C and D in cattle with different concentrations of recombinant proteins or the commercial vaccine until 180 days post-vaccination. Bars represent standard deviation. (**A**): Neutralizing antibody mean titers for each time point against BoNT serotype C; (**B**): Neutralizing antibody mean titers for each time point against BoNT serotype D.

The longevity of the immune response was measured through specific neutralizing antibody titers in each animal by seroneutralization assay in mice at days 0, 56, 120, and 180 (Table S1). On day 120 post-vaccination, neutralizing antibody mean titers were 0.625, 2.50, and 5.00 IU/mL for the respective different doses of anti-BoNT/C (Figure 4A), and 1.50, 3.75, and 4.00 IU/mL for anti-BoNT/D (Figure 4B). On the other hand, the commercial vaccine was able to produce mean titers of 1.25 and 0.50 IU/mL against BoNT serotypes C and D, respectively, resulting in significantly lower antibodies titers than the minimum required by the Brazilian legislation, with only 25% (2/8) of the sera with titers higher than the requested.

Animals that received doses containing 400 µg of recombinant protein were the only ones to achieve 100% (8/8) seroconversion for both toxins and overcome the minimum titers required by MAPA at day 120. When evaluating sera collected 180 days post vaccination, positive control group (commercial vaccine) and the group who received 100 mg of recombinant vaccine had no detectable levels of neutralizing antibodies against either toxin.

## 3. Discussion

Brazil has the 2nd largest cattle herd in the world and is the world’s largest beef exporter with more than 200 million bovines per year [21]. Since BoNTs are the most potent toxins known in nature and eradicating *C. botulinum* spores from the environment is virtually impossible, the best way to protect against botulism outbreaks in animals is through vaccination. At present, approximately 180 million doses of formaldehyde-inactivated botulism toxoids are produced annually in Brazil. Although these are effective on conferring protection, toxoids are time-consuming to prepare and are potentially hazardous during detoxification [11]. Therefore, the development of recombinant subunit vaccines has been evaluated as a mean of solving these problems [12,16,19,22,23].

To approve new commercial vaccines and ensure the quality, potency test for batches against botulism serotypes C and D in Guinea pigs are mandatory and should be done using a mice seroneutralization assay as per MAPA’s IN23 normative [20]. However, a tendency to obtain higher antitoxin titers in animal models than in the target animals (farm ruminants) has been observed in previous studies, both regarding the commercial toxoid vaccine and the recombinant protein vaccine [24,25]. In this study, only 75% of cattle vaccinated with commercial toxoid vaccine achieved satisfactory immune response at day 56, reinforcing the importance of assessing the immune status of vaccinated animals.

Neutralizing antibody titers were shown to be dependent on the dose of H_C_BoNT/C and D used in the vaccine. It was possible to establish a linear positive correlation between antigen amount per vaccine dose and neutralizing antibody titers for both BoNT serotypes C and D (r_C_^2^ = 0.823 and r_D_^2^ = 0.866) similar to correlation observed in buffaloes vaccinated with same recombinant vaccine (r_C_^2^ = 0.897 and r_D_^2^ = 0.920) [13]. These analyses show that at least 167 µg of H_C_BoNT/C and 108 µg of H_C_BoNT/D are required to induce a protective immune response in cattle, surpassing the minimum required by MAPA; likewise, 180 µg of H_C_BoNT/C and 105 µg of H_C_BoNT/D are necessary to achieve the same standards when vaccinating buffaloes [13].

Cunha et al. [12] were able to induce up to 5 and 6 IU/mL of antitoxins against BoNT serotypes C and D, respectively, in cattle immunized with 200 μg of a purified recombinant chimera composed by *Escherichia coli* heat-labile enterotoxin B subunit (LTB) and H_C_BoNT serotypes C and D (approximately 90 μg of each H_C_BoNT). In this study, cattle immunized with 100 µg of non-purified H_C_BoNT/C and D achieved mean titers of 1.25 and 1.5 IU/mL against serotypes C and D, respectively. Animals that were immunized with 200 µg per dose, produced up to 8 and 10 IU/mL of anti-BoNTs C and D, respectively, with 100% (8/8) seroconversion rate at day 56 post-vaccination. Furthermore, Otaka et al. [13] used the same strategy and dose to immunize buffaloes, obtaining 6.1 and 6.2 IU/mL of antitoxins against BoNTs C and D, respectively, in agreement with the requirements of MAPA’s normative instruction n. 23.

The highest neutralizing antibodies titers were achieved with 400 µg of H_C_BoNT serotypes C and D; also, 120 days post vaccination, this vaccine dose was also the only one that induced titers higher than the minimum required for protection against both serotypes in 100% of the animals. However, when evaluating sera collected 180 days post vaccination, none of tested doses was able to achieve the minimum mean titers based on the Brazilian normative.

Taken together, these results lead to the conclusion that 400 µg of non-purified recombinant H_C_BoNT are the best choice to induce the production of neutralizing antibodies against botulism serotypes C and D in cattle among the evaluated formulations. These formulations were capable of inducing higher antitoxin titers than those induced by the commercial toxoid used as control. Furthermore, the recombinant *E. coli* cell lysate supernatant dismisses the time-consuming and expensive steps for purification and detoxification. Thus, it is shown to be an affordable means of producing an effective, safe botulism vaccine for farm ruminants. Further studies still need to be performed focusing on adjuvant characterization, use of additional antigen vehicles, or changing vaccination protocols to achieve high titers of neutralizing antibodies in cattle capable of conferring protection for, ideally, at least 12 months.

## 4. Materials and Methods

### 4.1. Vaccine Formulation and Safety

Vaccine formulations containing whole-cell lysates were prepared as previously described [19], with minor modifications to allow soluble expression of H_C_BoNT/C, which was insoluble in previous works. Bivalent recombinant vaccines were obtained from the cell lysate supernatant and contained 100, 200 or 400 µg of non-purified H_C_BoNT/C and D per dose and aluminum hydroxide as adjuvant in a final volume of 5 mL. Vaccine sterility was evaluated by inoculating thioglycolate and Sabouraud broths at 37 °C and 25 °C, respectively, and checking for growth daily for 21 days by spectrophotometry. Innocuity was assessed by inoculating two cattle with twice the vaccine dose (800 µg) and observing for side effects for 72 h. These protocols are described elsewhere [20].

### 4.2. Cattle Vaccination

Forty 18-month old Nellore cattle raised on pasture were randomly segregated into 5 groups of 8 animals each. Animals had food and water ad libitum and were handled equally. All animals had no detectable antibody against BoNT serotypes C and D measured through seroneutralization assay in mice prior to the beginning of the experiment. Vaccines were administered on days 0 and 28; blood was taken from the jugular vein at days 0, 56, 120, and 180 with serum samples obtained by centrifugation (3000× *g*, 15 min) and stored at −20 °C until use.

Groups 1, 2, and 3 were vaccinated with 100, 200, and 400 µg of each recombinant protein, respectively, adsorbed onto aluminium hydroxide in a final volume of 5 mL per dose. Group 4 received a bivalent commercial vaccine against BoNT serotypes C and D, following manufacturer’s instructions, as a positive control. Group 5 was inoculated with sterile NaCl solution (0.9% *w*/*v*) and aluminium hydroxide as a negative control. All animal experiments were performed in accordance with the guidelines of the National Council for Animal Experimentation Control (CONCEA) and the Ethics Committee in Animal Experimentation of the Federal University of Pará (CEEA-UFPA). In terms of the latter, the project was approved and completed under permit No. 9668220616 (08/25/2016).

### 4.3. Serum Neutralization Assay

Cattle sera were evaluated individually using the serum neutralization bioassay in mice as described by MAPA in its normative instruction number 23 (NI 23) [20]. The procedures were based on the European Pharmacopoeia and on the Code of Federal Regulations Title 9 (CFR 9, USDA) for measuring BoNTs/C and D antitoxins. Briefly, 1 mL of each toxin (produced by LANAGRO (Pedro Leopoldo, MG, Brazil) and standardized using antibodies provided by NIBSC (Potters Bar, United Kingdom) was incubated at 37 °C for 1 h with 1 mL of 2-fold serial dilutions of each serum from 1:1 to 1:32. Then, two Swiss Webster mice weighing 18–22 g were intravenously inoculated with 0.2 mL of each sample per dilution and observed for 72 h for survival and euthanized if necessary. Later on, the procedure was repeated with intermediary dilutions of each serum to identify the exact protective titer. The survival information was used to calculate the results in international units per milliliter (IU/mL). The lowest required titers established in the aforementioned references were considered to be the minimum protective titers in this study.

### 4.4. Statistics Analysis

Minitab® v.17.1.0 statistical software (Minitab Inc., PA, USA, 2013) was used to perform analysis of variance (ANOVA) and Tukey’s post-test to identify significant differences in antibody titers among the groups. A regression analysis was conducted for the concentration of recombinant proteins and titers against BoNTs C and D.

## Figures and Tables

**Table 1:** Titers of neutralizing antibodies against botulinum neurotoxin (BoNT) serotypes C and D at day 56 post vaccination for individual animals vaccinated with commercial vaccine (CV) and different concentrations of the recombinant vaccine (100, 200 and 400 µg). This was tested by the serum neutralization bioassay in mice.

	Treatment	Anti-BoNT Serotype C titers (IU/mL)					Anti-BoNT Serotype D titers (IU/mL)				
Animal		100 µg	200 µg	400 µg	CV	PBS	100 µg	200 µg	400 µg	CV	PBS
Animal 1		ND ^a^	8	10	5	ND	2	10	15	3	ND
Animal 2		5	10	10	5	ND	ND	10	15	2	ND
Animal 3		ND	8	12	ND	ND	2	10	15	ND	ND
Animal 4		ND	10	12	6	ND	ND	10	15	5	ND
Animal 5		ND	10	12	5	ND	ND	12	15	ND	ND
Animal 6		5	10	12	6	ND	4	10	15	4	ND
Animal 7		ND	8	10	5	ND	2	12	10	2	ND
Animal 8		ND	5	10	ND	ND	2	8	10	5	ND
Mean ± SD ^b^		1.25 ± 2.32	8.65 ± 1.77	11.0 ± 1.07	4.0 ± 2.51	0	1.5 ± 1.41	10.25 ± 1.28	13.75 ± 2.32	2.62 ± 2.0	0
Seroconversion Rate ^c^		25%	100%	100%	75%		62.5%	100%	100%	75%	

^a^ ND, non-detectable levels of neutralizing antibodies. ^b^ Standard deviation. ^c^ Consideration of the minimum antibodies titers required by NI 23 Brazilian Ministry of Agriculture, Livestock and Food Supply (MAPA) [20].

## Supplementary Materials

The following are available online at http://www.mdpi.com/2072-6651/10/10/381/s1, Table S1: Titers of neutralizing antibodies against BoNT serotypes C and D at day 0, 56, 120, and 180 post vaccination for individual animals vaccinated with commercial vaccine (CV) and different concentrations of the recombinant vaccine (100, 200 and 400 µg).
