# Supplementary material for: Immunogenicity of a Bivalent Non-Purified Recombinant Vaccine against Botulism in Cattle
Source: Toxins (Basel). 2018 Sep 20;10(10):381. doi: 10.3390/toxins10100381 (PMC6215264; doi:10.3390/toxins10100381)
Supplement: Supplementary file 1 [file toxins-10-00381-s001.pdf]

Supplementary Materials: Immunogenicity of A Bivalent Non-Purified Recombinant Vaccine Against Botulism in Cattle

Clóvis Moreira, Jr., Marcos R. A. Ferreira, Carlos E. P. da Cunha, Rafael A. Donassolo, Paula F. Finger, Gustavo M.S.G. Moreira, Denis Y. Otaka, Loise A. de Sousa, José D. Barbosa, Ângela N. Moreira, Felipe M. Salvarani and Fabricio R. Conceição

Table S1. Titers of neutralizing antibodies against BoNT serotypes C and D at days 0, 56, 120, and 180 post vaccination for individual animals vaccinated with Commercial vaccine (CV) and different concentrations of the recombinant vaccine (100, 200 and 400 μg). These values were obtained with the serum neutralization bioassay in mice.

		Anti-BoNT/C (IU/mL)				Anti-BoNT/D (IU/mL)			
		**Day 0**	**Day 56**	**Day 120**	**Day 180**	**Day 0**	**Day 56**	**Day 120**	**Day 180**
100 μg Hc-BoNT/C and D	Animal 1	ND	ND[1]	ND	ND	ND	2	2	ND
	Animal 2	ND	5	5	ND	ND	ND	ND	ND
	Animal 3	ND	ND	ND	ND	ND	2	2	ND
	Animal 4	ND	ND	ND	ND	ND	ND	ND	ND
	Animal 5	ND	ND	ND	ND	ND	ND	ND	ND
	Animal 6	ND	5	5	ND	ND	4	4	ND
	Animal 7	ND	ND	ND	ND	ND	2	2	ND
	Animal 8	ND	ND	ND	ND	ND	2	2	ND
200 μg Hc-BoNT/C and D	Animal 1	ND	8	ND	ND	ND	10	4	ND
	Animal 2	ND	10	5	ND	ND	10	4	ND
	Animal 3	ND	8	ND	ND	ND	10	4	ND
	Animal 4	ND	10	5	5	ND	10	4	2
	Animal 5	ND	10	5	ND	ND	12	4	ND
	Animal 6	ND	10	5	ND	ND	10	4	ND
	Animal 7	ND	8	ND	ND	ND	12	4	ND
	Animal 8	ND	5	ND	ND	ND	8	2	ND
400 μg Hc-BoNT/C and D	Animal 1	ND	10	5	ND	ND	15	4	ND
	Animal 2	ND	10	5	ND	ND	15	4	ND
	Animal 3	ND	12	5	ND	ND	15	4	ND
	Animal 4	ND	12	5	5	ND	15	4	2
	Animal 5	ND	12	5	ND	ND	15	4	ND
	Animal 6	ND	12	5	ND	ND	15	4	2
	Animal 7	ND	10	5	ND	ND	10	4	2
	Animal 8	ND	10	5	5	ND	10	4	ND
Commercial vaccine	Animal 1	ND	5	ND	ND	ND	3	ND	ND
	Animal 2	ND	5	ND	ND	ND	2	ND	ND
	Animal 3	ND	ND	ND	ND	ND	ND	ND	ND
	Animal 4	ND	6	5	ND	ND	5	2	ND
	Animal 5	ND	5	ND	ND	ND	ND	ND	ND
	Animal 6	ND	6	5	ND	ND	4	ND	ND
	Animal 7	ND	5	ND	ND	ND	2	2	ND

	Animal 8	ND	ND	ND	ND	ND	5	ND	ND
Negative control	Animal 1	ND	ND	ND	ND	ND	ND	ND	ND
	Animal 2	ND	ND	ND	ND	ND	ND	ND	ND
	Animal 3	ND	ND	ND	ND	ND	ND	ND	ND
	Animal 4	ND	ND	ND	ND	ND	ND	ND	ND
	Animal 5	ND	ND	ND	ND	ND	ND	ND	ND
	Animal 6	ND	ND	ND	ND	ND	ND	ND	ND
	Animal 7	ND	ND	ND	ND	ND	ND	ND	ND
	Animal 8	ND	ND	ND	ND	ND	ND	ND	ND

[1] ND, non detectable levels of neutralizing antibodies.
